# Extracellular Ig_C2_ Constant Domains of CEACAMs Mediate PI3K Sensitivity during Uptake of Pathogens

**DOI:** 10.1371/journal.pone.0039908

**Published:** 2012-06-29

**Authors:** Maike Voges, Verena Bachmann, Jan Naujoks, Kathrin Kopp, Christof R. Hauck

**Affiliations:** 1 Lehrstuhl Zellbiologie, Universität Konstanz, Konstanz, Germany; 2 Konstanz Research School Chemical Biology, Universität Konstanz, Konstanz, Germany; Columbia University, United States of America

## Abstract

**Background:**

Several pathogenic bacteria utilize receptors of the CEACAM family to attach to human cells. Binding to different members of this receptor family can result in uptake of the bacteria. Uptake of *Neisseria gonorrhoeae*, a Gram-negative human pathogen, via CEACAMs found on epithelial cells, such as CEACAM1, CEA or CEACAM6, differs mechanistically from phagocytosis mediated by CEACAM3, a CEACAM family member expressed selectively by human granulocytes.

**Principal Findings:**

We find that CEACAM1- as well as CEACAM3-mediated bacterial internalization are accompanied by a rapid increase in phosphatidylinositol-3,4,5 phosphate (PI(3,4,5)P) at the site of bacterial entry. However, pharmacological inhibition of phosphatidylinositol-3′ kinase (PI3K) selectively affects CEACAM1-mediated uptake of *Neisseria gonorrhoeae*. Accordingly, overexpression of the PI(3,4,5)P phosphatase SHIP diminishes and expression of a constitutive active PI3K increases CEACAM1-mediated internalization of gonococci, without influencing uptake by CEACAM3. Furthermore, bacterial uptake by GPI-linked members of the CEACAM family (CEA and CEACAM6) and CEACAM1-mediated internalization of *N. meningitidis* by endothelial cells require PI3K activity. Sensitivity of CEACAM1-mediated uptake toward PI3K inhibition is independent of receptor localization in cholesterol-rich membrane microdomains and does not require the cytoplasmic or the transmembrane domain of CEACAM1. However, PI3K inhibitor sensitivity requires the Ig_C2_-like domains of CEACAM1, which are also present in CEA and CEACAM6, but which are absent from CEACAM3. Accordingly, overexpression of CEACAM1 Ig_C2_ domains blocks CEACAM1-mediated internalization.

**Conclusions:**

Our results provide novel mechanistic insight into CEACAM1-mediated endocytosis and suggest that epithelial CEACAMs associate in *cis* with other membrane receptor(s) via their extracellular domains to trigger bacterial uptake in a PI3K-dependent manner.

## Introduction

Carcinoembryonic antigen-related cell adhesion molecule 1 (CEACAM1) is a widely expressed glycoprotein of the CEACAM family, which in humans comprises 12 genes and a number of pseudogenes [1], [2]. Similar to several other human CEACAMs, CEACAM1 is characterized by an amino-terminal immunoglobulin variable-like domain (Ig_V_-like), followed by one to three immunoglobulin constant type2 (Ig_C2_)-like domains, which are defined by the reduced number of beta-strands in the Ig fold compared to Ig_V_-like domains [3]. CEACAM1 is involved in a broad spectrum of cellular processes ranging from tissue morphogenesis and apoptosis, to insulin homeostasis, angiogenesis, or regulation of T-cell activity [2]. Another member of the CEACAM family is CEACAM3, which is exclusively expressed on granulocytes and harbours a single Ig_V_-like domain followed by a transmembrane helix and a cytoplasmic domain [4]. Besides CEACAM1 and CEACAM3, two additional members of the CEACAM family, namely CEA (the product of the *CEACAM5* gene) and CEACAM6, can serve as cellular receptors for a range of gram-negative bacteria [5]–[11]. In all these cases, bacteria engage the non-glycosylated face of the N- terminal Ig_V_-like domain, which shares more than 90% sequence similarity between the four CEACAMs exploited as host receptors.

A common hallmark of CEACAM-binding bacteria, such as *Neisseria gonorrhoeae*, *N. meningitidis*, *N. lactamica* or *Moraxella catarrhalis*, is their ability to efficiently colonize mucosal epithelia of the human body. Indeed, engagement of epithelial CEACAM family members, such as CEACAM1, CEA, or CEACAM6, allows the microorganisms to trigger increased matrix-adhesion of the infected host cells [12]. For *N. gonorrhoeae*, this process promotes mucosal colonization in vivo by preventing exfoliation of superficial epithelial cells from stratified tissues in the urogenital tract [13]. Furthermore, the binding to CEACAMs can trigger bacterial engulfment by various cell types including both professional as well as non-professional phagocytes [7], [14]–[18]. Interestingly, in polarized epithelia CEACAMs are generally expressed on the apical membrane and support not only internalization, but also transcytosis of CEACAM-binding bacteria through an intact cell layer [19], [20]. How internalization and transcellular trafficking via epithelial CEACAMs is regulated on the molecular level is currently unclear.

In contrast to epithelial CEACAMs, the endocytotic function of CEACAM3, a bacteria-binding member of the family exclusively expressed on granulocytes, has been delineated in great detail (for review see [21]). The cytoplasmic domain of CEACAM3 contains a sequence with similarity to the so-called immunoreceptor tyrosine-based activation motif (ITAM), which is phosphorylated by Src family protein tyrosine kinases upon bacterial engagement [22]. The phosphorylated cytoplasmic domain of CEACAM3 coordinates the local assembly of a signalling complex, which is responsible for the efficient internalization of bound bacteria [23]–[25]. By directly associating with the SH2 domains of the guanine nucleotide exchange factor (GEF) Vav and the adaptor molecule Nck, CEACAM3 recruits an upstream stimulator of the small GTPase Rac (the GEF Vav) and a downstream effector of GTP-loaded Rac (the Nck-associated WAVE-complex) to trigger massive actin rearrangements required for CEACAM3-mediated phagocytosis [26], [27]. The involvement of the CEACAM3 cytoplasmic domain and the strict requirement for dynamic actin rearrangements is clearly distinct from epithelial CEACAMs, which mediate endocytosis in the absence of a cytoplasmic domain. Indeed, epithelial CEACAMs either do not possess cytoplasmic domains (such as the GPI-anchored CEA and CEACAM6) or their endocytotic function is not affected by the deletion of the cytoplasmic domain (as is the case for CEACAM1) [22], [28], [29]. Moreover, interference with actin polymerization only partially affects endocytosis via epithelial CEACAMs [22], [30]. Interestingly, epithelial CEACAMs re-locate into sphingolipid-rich membrane microdomains upon clustering, and cholesterol-depletion affects their internalization, whereas CEACAM3-mediated uptake is not sensitive to cholesterol-chelators [22], [28]. Together, the previous studies suggest that internalization initiated by epithelial CEACAMs (CEACAM1, CEA and CEACAM6) is mechanistically distinct from CEACAM3-triggered phagocytosis, though they share the same ligands. Besides the distribution into membrane microdomains, which for CEACAM1 seems to depend on determinants in the transmembrane helix, no further downstream factors responsible for the distinct behaviour of epithelial CEACAMs during endocytosis have been described.

Recently, we made the surprising observation that phosphatidylinositol-3′ kinase (PI3Ks) activity is not involved in CEACAM3-triggered phagocytosis by human granulocytes [31], despite the well-characterized role of phosphatidylinositol phosphates (PIPs) in numerous endocytic processes [32], [33]. These findings prompted us to investigate the involvement of PI3K and PIPs in bacterial internalization via epithelial CEACAMs. We report here, that 3′-phosphorylated PIPs are not only enriched around bacterial uptake sites, but that blockade of PI3Ks severely reduces bacterial internalization via CEACAM1 and other epithelial CEACAMs. In line with a role of phosphatidylinositol 3,4,5-phosphate (PI(3,4,5)P), overexpression of class I PI3K increases, whereas expression of SHIP, which dephosphorylates PI(3,4,5)P, reduces CEACAM1-mediated uptake. Surprisingly, PI3K-dependent endocytosis of CEACAM1 was not connected to cytoplasmic determinants of the receptor or to its location in membrane microdomains, but rather required the extracellular Ig_C2_-like domains of CEACAM1. These data provide novel mechanistic insight into CEACAM1 endocytosis and suggest the existence of *cis*-interactions between epithelial CEACAMs and additional membrane proteins triggering PI3K-dependent uptake.

## Materials and Methods

### Neisserial strains and growth conditions

Opa_52_ protein-expressing, non-piliated *Neisseria gonorrhoeae* strain MS11 (Ngo Opa_CEA_) and the isogenic non-opaque strain (Ngo Opa-) were kindly provided by Thomas F. Meyer (Max-Planck-Institut für Infektionsbiologie, Berlin, Germany). Opa_CEA_ protein-expressing, unencapsulated *Neisseria meningitidis* strain MC58 (ΔsiaD, ΔlgtA) (Nm Opa_CEA_) was obtained from Matthias Frosch (Institut für Hygiene und Mikrobiologie, Universität Würzburg, Germany). Both, *Neisseria meningitidis* and *N. gonorrhoeae* were grown as described before [34] on GC agar plates (Difco BRL, Paisley, UK) supplemented with vitamins at 37°C, 5% CO_2_. For infection, over-night grown bacteria were taken from GC agar plates, suspended in PBS, and colony forming units (cfu) were estimated by OD_550_ readings according to a standard curve.

### Epithelial and endothelial cell lines

Human embryonic kidney epithelial 293T cells (293 cells; ACC-635, DSMZ, Braunschweig, Germany) were cultured in Dulbecco's modified Eagle's medium (DMEM) containing 10% calf serum. Human brain microvascular endothelial cells (HBMEC) [35] were grown in endothelial cell medium (PAA, Pasching, Austria) supplemented with L-glutamine. All cells were grown in the absence of antibiotics at 37°C in 5% CO_2_ and subcultured every 2–3 days.

### Recombinant DNA constructs

Mammalian expression plasmids encoding HA-tagged versions of human CEACAM1-4L (CEACAM1), CEACAM1 lacking the complete cytoplasmic domain (CEACAM1-ΔCT), CEACAM3, CEA and CEACAM6 were described previously [12], [24]. The mKate-tagged and mCerulean-tagged versions of CEACAM1, CEACAM1-ΔCT, and CEACAM3 were generated by amplifying the HA-tagged versions of CEACAM1, CEACAM1-ΔCT, or CEACAM3, respectively, with primers CEACAM1-IF sense 5′-GAAGTTATCAGTCGATACCATGGGGCACTTCTCAGCCCC-3′ and HA-CEACAM-IF antisense 5′-ATGGTCTAGAAAGCTTGCAGCGTAATCTGGAACGTCATATGG-3′, or with CEACAM3-IF-sense, 5′-GAAGTTATCAGTCGATACCATGGGGCCCCCCTCAGCC-3′, and HA-CEACAM-IF-antisense. The resulting PCR fragments were cloned into pDNR-Dual using the In-Fusion PCR Cloning Kit (Clontech, Mountain View, CA) and transferred by Cre-mediated recombination into pLPS-3′mKate [13] or pLPS-3′mCerulean [11] resulting in mKate or mCerulean, respectively, fused to the carboxy-terminus of the expressed proteins. The different CEACAM1 deletion mutants lacking either three, two or one Ig_C2_-like extracellular domains (CEACAM1-N, CEACAM1-NA1, CEACAM1-NA1B) and the CEACAM8/1 chimera were described previously [36]. The CEACAM1/CEACAM3-TM chimera was constructed by amplifying CEACAM1 lacking the cytoplasmic domain with sense-Chimera3cd-HA primer 5′-ATAATGGCCATAGTGGCGCTGGTGGCCGCACTGGTGTGTTTCCTGCTCCTTCATTTCGGGAAATATCCCTATGACG-3′ and rev-Chimera3ab primer 5′-ATAATGGCCACTCCGACCAGGACCCCGGTCACGATCCCAGGTGAGAGGCC-3′ resulting in the CEACAM1 extracellular domains fused to the transmembrane domain of CEACAM3. The plasmids pEGFP-Btk-PH and pEGFP-PLCδ-PH were a kind gift from T. Balla (NIH, Bethesda, MD).

The cDNA of the enzymatic p110 subunit of PI3K was a gift from J. Downward (Cancer Research UK, London, UK). Full-length PI3K was amplified with primers PI3KCA-IF-sense-5′- GAAGTTATCAGTCGACCCTCCAAGACCATCATCAG-3′ and PI3KCA-IF-anti -5′-ATGGTCTAGAAAGCTTAGGCGGCTCAGTTCAATGCATGCTG-3′. The resulting PCR fragment was cloned into pDNR-Dual using the In-Fusion PCR Cloning Kit (Clontech, Mountain View, CA) and transferred by Cre-mediated recombination into a modified pcDNA vector with loxP site 3′ of the cerulean coding sequence (pcDNA Cerulean loxP). The cDNAs of murine SHIP1 and the phosphatase inactive mutant SHIP D675G were a kind gift from G. Krystal (British Columbia Cancer Agency, Vancouver, Canada). The phosphatase domains were cloned in pDNR-dual with primers SHIP-PD-IF sense-5′ GAAGTTATCAGTCGACGAGCCAGAGCCTGAC-3′ and SHIP-PD-IF antisense-5′ ATGGTCTAGAAAGCTTAAGGGACCCTGCCAGAAGG-3′ and transfered in pcDNA Cerulean loxP via Cre-mediated recombination.

The N-terminal SH2 domains of PI3KR3 and Hck were previously described [31]. The C-terminal SH2 domain of SHP2 was constructed by amplification from human SHP2 cDNA (a gift from B.G. Neel, University of Toronto, Toronto, Canada) with primers PTPN11-C-SH2-IF-sense 5′-GAAGTTATCAGTCGACCCTCTGAACTGTGCAGATC-3′ and PTPN11-C-SH2-IF-anti 5′-ATGGTCTAGAAAGCTTAATTTATACGAGTCGTGTTAAG-3′. The resulting PCR fragments were cloned into pDNR-Dual using the In-Fusion PCR Cloning Kit (Clontech, Mountain View, CA) and transferred by Cre-mediated recombination into pGEX4-T1 loxP [26]. The cloning and production of CEACAM1-GFP or GFP encoding lentiviral particles according to Rubinson et al. [37] and transduction of cells was performed as described previously [29].

### Antibodies and reagents

Monoclonal antibody (mAb) against CEACAMs (clone D14HD11; recognizing CEACAM1, CEACAM3, CEA and CEACAM6) were purchased from Genovac (Freiburg, Germany), mAb against Flotillin2 (clone ESA) was from BD Biosciences (Heidelberg, Germany), mAbs against the HA-tag (clone 12CA5), against tubulin (clone E7), and against transferrin (clone G1/221/12) were purified from hybridoma cell supernatants. Rabbit polyclonal α-GFP antibody was custom made at our in-house Animal Research Facility (Universität Konstanz, Germany) and affinity purified. Secondary antibodies were purchased from Jackson ImmunoResearch (West Grove, PA).

### Transfection of cells, cell lysis, GST pulldown assay and Western blot

293 cells were transfected by calcium phosphate precipitation using 5–8 µg of appropriate cDNA in each case. The transfection efficiency ranged between 30% and 70% as reported [22]. Transfected 293 cells were employed in experiments 48 h after transfection. Cell lysis, Western blotting, and GST pulldown assays were performed as described [24], [31].

### Gentamicin protection assay

293 cells were seeded at 5×10^5^ cells/well in 24-well plates or 2.5×10^5^ cells/well in 48-well plates coated with poly-L-lysin (10 µg/ml). A multiplicity of infection (MOI) of 30 bacteria per cell was routinely used, and after 2 hour of infection, extracellular bacteria were killed by 60 min incubation in 50 µg/ml gentamicin in DMEM. In the case of endothelial cells, HBMECs were seeded at 2×10^5^ cells/well in 24-well plates coated with poly-L-lysin (10 µg/ml). A multiplicity of infection (MOI) of 40 bacteria was used, and after 3 hour of infection, extracellular bacteria were killed by 60 min incubation in 50 µg/ml gentamicin in endothelial medium. Following gentamicin treatment, cells were lysed with 1% saponin in PBS for 15 min. The samples were diluted in PBS, and the number of viable bacteria was determined by plating suitable dilutions on GC agar. For inhibition studies, cells were treated with the inhibitors wortmannin (200 nM), LY294002 (50 µM) or methyl-β-cyclodextrin (500 µM) 30 min prior to infection.

### Bacterial adherence assay

Cells were seeded and infected as described for gentamicin protection assays. After the infection, the cells were gently washed three times, before they were lysed by addition of 1% saponin in PBS for 15 min. Total cell-associated bacteria were suspended by vigorous pipetting, and colony forming units were determined by plating of serial dilutions on GC agar.

### Flow cytometry-based invasion assay

Bacterial uptake by 293 cells was analysed by flow cytometry as described [38]. Prior to infection, bacteria were surface labelled with 0.2 µg/ml 5-(6)-carboxyfluorescein-succinimidylester (fluorescein; Invitrogen-Molecular Probes, Karlsruhe, Germany) in PBS at 37°C for 30 min prior to infection. 1×10^6^ cells were seeded in 6-well plates coated with poly-L-lysin (10 µg/ml) and the next day, cells were infected with labelled bacteria at an MOI of 30 for 2 h. After infection, cells were washed with PBS and the samples were analysed on a LSR II flow cytometer (BD Bioscience) by gating on the cells based on forward and sideward scatter. Cell-associated fluorescein fluorescence was measured in the presence of 2 mg/ml trypan blue to quench fluorescence derived from extracellular bacteria and to selectively detect the fluorescence derived from intracellular bacteria. The percentage of fluorescein-positive cells within the transfected cell population was multiplied by the mean fluorescence intensity of the sample to obtain an estimate of the total number of internalized bacteria (uptake index). In each sample at least 4,000 cells were counted.

### Immunofluorescence staining

For microscopic analysis of 293 cells, 7×10^4^ cells were seeded onto poly-L-lysine- and fibronectin-coated (10 and 4 µg/ml, respectively, in PBS) glass-coverslips in 24-well plates. For microscopic analysis of HBMEC cells, 3×10^4^ cells were seeded onto poly-L-lysine- and fibronectin-coated (10 and 4 µg/ml, respectively, in PBS) glass-coverslips in 24-well plates. Cells were infected with Pacific blue-labeled (and in case of intra-/extracellular staining additionally biotinylated) bacteria for 3 h at an MOI of 30 (HBMEC with meningococci) or for 1 h at an MOI of 40 (293 cells with gonococci). Samples were washed once and fixed with 4% paraformaldehyde. After three washes with PBS, samples were incubated in blocking buffer (PBS, 10% fetal calf serum) for 5 min and then stained for extracellular bacteria with streptavidin-Cy3 (diluted 1∶100 in blocking buffer). Following three washes, samples were embedded in mounting medium (Dako, Glostrup, Denmark). Samples were analysed with a Leica TCS SP5 confocal laser scanning microscope (Leica Microsystems, Wetzlar, Germany). Fluorescence signals of triple-labelled specimens were serially recorded with appropriate excitation and emission filters to avoid bleed-through. Images were digitally processed with ImageJ and merged to yield pseudo-coloured pictures.

### Analysis of detergent-resistant membrane fractions

To analyse the association of proteins with detergent-resistant membrane (DRM) fractions, the flotation assay as described by Umlauf et al. [39] was used with slight modifications. Briefly, 293 cells were transfected with HA-tagged recombinant constructs of CEACAM1 or CEACAM3 together with GFP-tagged flotilin2. Transfected cells were left untreated or CEACAMs were clustered as described [28]. In some cases, transfected cells were treated with 200 nM wortmannin prior to CEACAM. After washing with ice-cold PBS, cells were homogenized in ice-cold buffer containing 50 mM Tris, pH 7.4, 2 mM MgCl_2_ and 8% sucrose and centrifuged for 15 min at 5700 *g*. The pellet of unbroken cells and organelles was discarded and the supernatant was adjusted to 4 mM EDTA, before centrifugation at 48 000 rpm for 2 h using an SW60 rotor (Beckman). The resulting pellet containing cellular membranes was subsequently solubilized at 4°C for 15 min in 280 µl of ice-cold TNE-T buffer (20 mM Tris-Cl, pH 8.0, 130 mM NaCl, 5 mM EDTA, 0.7% Triton X-100 and protease inhibitors (10 µg ml^−1^ aprotinin and leupeptin, 1 µg ml^−1^ pepstatin A and 1 mM phenylmethyl-sulfonyl fluoride). The lysate was adjusted to 50% sucrose by the addition of 490 µl of 80% sucrose in TNE-T and covered with 3 ml of 35% sucrose followed by 1.2 ml of 5% sucrose in the same buffer. After ultracentrifugation at 48 000 rpm in a SW 60 rotor (Beckman) for 18 h, eight fractions were collected from the top of the gradient and analysed by Western blotting. DRM components were recovered in the low-density fractions 2–4.

## Results

### Phosphatidylinositol 3,4,5-phosphate is generated during CEACAM1- and CEACAM3-mediated bacterial host cell contact

**Figure 1:**
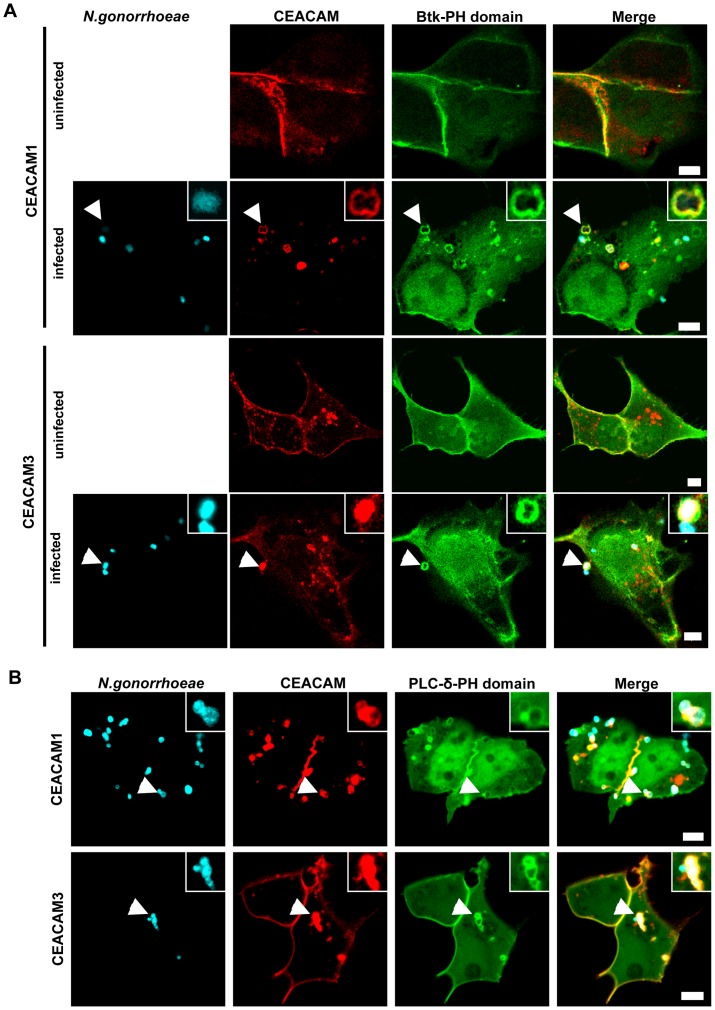
PI(3,4,5)P and PI(4,5)P are generated during CEACAM1- and 3- mediated bacterial entry. (**A**) 293 cells were cotransfected with constructs encoding mKate-tagged CEACAM1-4L or CEACAM3 together with GFP-tagged Btk-PH domain (which binds specifically to PI(3,4,5)P). Cells were left uninfected or were infected with Pacific Blue labelled Opa_CEA_-expressing *N. gonorrhoeae* for 60 min, fixed and analysed by confocal microscopy. Arrowheads highlight bacteria associated with CEACAMs and the Btk-PH domain. Insets show enlargement of the highlighted area. Bars represent 5 µm. (**B**) Cells were cotransfected with CEACAM constructs as in A) together with the GFP-tagged PLC-δ-PH domain (which specifically binds to PI(4,5)P). Cells were infected and analyzed as in (A). Arrowheads highlight bacteria associated with CEACAMs and the PLC-δ-PH domain. Insets show enlargement of the highlighted area. Bars represent 5 µm.

It is well documented, that PI(3,4,5)P constitutes a critical host factor during endocytosis of bacteria via distinct surface receptors [40]. However, we observed previously that lipid kinases of the PI3K family, which generate PI(3,4,5)P, are not required for bacterial uptake via CEACAM3 [31]. To investigate, if PI(3,4,5)P might be involved in CEACAM1-mediated internalization, we co-transfected 293 cells with constructs encoding GFP-tagged PH domain of Bruton's tyrosine kinase (Btk) together with either mKate-tagged CEACAM1 or mKate-tagged CEACAM3. The PH-domain of Btk can serve as an intracellular PI(3,4,5)P reporter, as it binds specifically to this membrane lipid [41]. The transfected cells were either left uninfected or were infected for 60 min with Pacific Blue-labelled *N. gonorrhoeae* expressing a CEACAM-binding Opa protein (Ngo Opa_CEA_). After infection, the samples were fixed and analyzed by confocal microscopy (Fig. 1A). Whereas the PH domain was evenly distributed in the membrane of uninfected cells, engagement of CEACAM3 as well as CEACAM1 by gonococci resulted in a massive recruitment of the Btk-PH domain to the site of bacteria-host cell contact (Fig. 1A). PI(3,4,5)P can be generated from PI(4,5)P by the action of class I PI3Ks. To address, if the class I PI3K substrate is present at the site of bacterial uptake, we co-transfected cells with CEACAM1-mKate or CEACAM3-mKate expression vectors together with a construct encoding the GFP-tagged PH-domain of PLC-δ, which binds specifically to PI(4,5)P [41]. Similar to the recruitment of Btk-PH domain, bacterial engagement of either CEACAM resulted in a pronounced accumulation of the PLC-δ-PH domain (Fig. 1B). Together, these results indicate that both the substrate (PI(4,5)P) as well as the product (PI(3,4,5)P) of class I PI3Ks accumulate upon binding of bacteria to CEACAM1 or CEACAM3.

### PI3K inhibition selectively interferes with uptake of Opa_CEA_-expressing gonococci via CEACAM1

**Figure 2:**
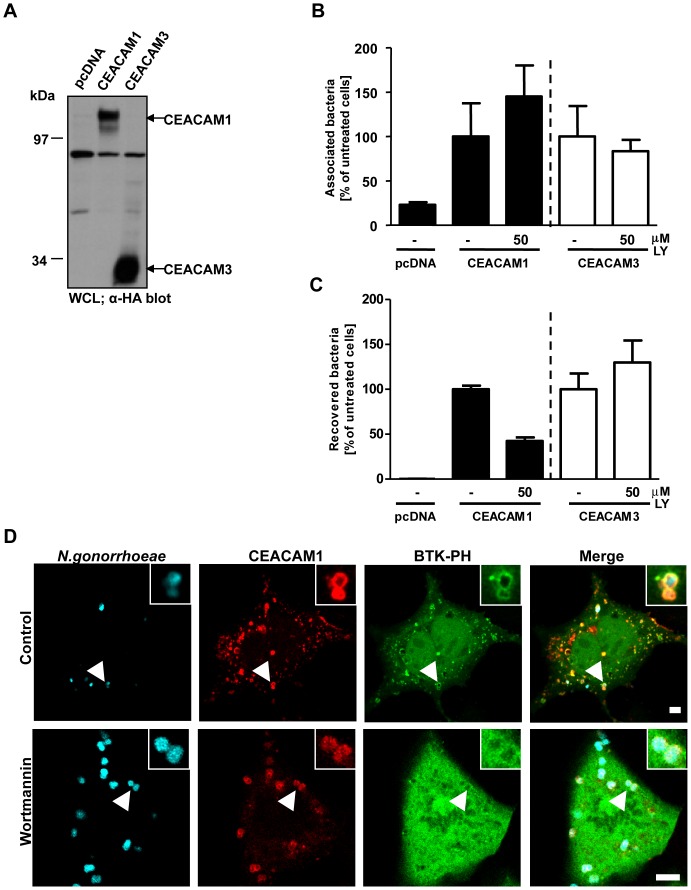
Inhibition of PI3K activity decreases uptake of Opa_CEA_-expressing gonococci via CEACAM1. (**A**) 293 cells were transfected with empty vector pcDNA, CEACAM1-4L or CEACAM3 WT. CEACAM expression was verified by Western blotting of whole cell lysates (WCL) using α-HA antibody. (**B**) Cells were transfected as in (A) and pretreated for 30 min with 50 µM of the PI3K inhibitor LY294002 (LY). After infection for 2 h with Opa_CEA_-expressing gonococci, the number of total cell-associated bacteria was determined. Bars represent mean values ± S.E.M of three independent experiments done in triplicate. Total cell associated bacteria are shown relative to cells expressing the respective receptor without PI3K inhibitor treatment. (**C**) Cells were transfected and infected as in (B). Viable intracellular bacteria were determined in gentamicin protection assays. Bars represent mean values ± S.E.M of three independent experiments done in triplicate. Recovered bacteria are shown relative to cells expressing the respective receptor without PI3K inhibitor treatment. (**D**) 293 cells were cotransfected with mKate-tagged CEACAM1 and the GFP-tagged Btk-PH domain. 30 min before infection with Pacific Blue labelled Opa_CEA_-expressing gonococci, cells were treated with 200 nM wortmannin or left untreated. Fixed samples were analyzed by confocal microscopy. Arrowheads highlight CEACAM- recruitment to cell-associated bacteria. Insets show enlargement of the highlighted area. Bars represent 5 µm.

Since the PI(3,4,5)P level in unstimulated cells is generally low, the accumulation of this phospholipid around CEACAM-bound bacteria suggested the local activation of class I PI3Ks. Though PI3Ks are not involved in CEACAM3-mediated internalization, we wondered whether these enzymes play a role during CEACAM1-mediated uptake of bacteria. Therefore, we transfected cells with constructs encoding HA-tagged CEACAM1 or CEACAM3 or the empty vector and similar expression was verified by Western blotting with a monoclonal antibody against the HA-tag (Fig. 2A). Then, the activity of endogenous PI3K in CEACAM1- or CEACAM3 transfected cells was blocked by addition of the PI3K selective inhibitor LY294002, before cells were infected with Opa_CEA_-expressing gonococci for 2 h. Following the infection, cells were washed and total cell associated bacteria were enumerated by plating dilutions on selective media (adherence assay; Fig. 2B). Parallel samples were treated with gentamicin for 1 hour to kill extracellular bacteria, and the intracellular bacteria were then released by mild detergent lysis of the eukaryotic cells (invasion assay; Fig. 2C). Importantly, cells transfected with the empty vector did only associate with low numbers of bacteria and did not contain viable intracellular bacteria (Fig. 2B and C). In contrast, transfection with either CEACAM1 or CEACAM3 allowed cells to associate with Opa_CEA_-expressing *N. gonorrhoeae* (Fig. 2B). Incubation with LY294002 did not decrease binding of the bacteria to CEACAMs and even slightly increased cell-associated bacteria in CEACAM1-expressing cells (Fig. 2B). Whereas CEACAM3-mediated uptake was not reduced in the presence of the PI3K inhibitor, a finding that corroborates previous results [31], bacterial internalization via CEACAM1 was clearly diminished by LY294002 (Fig. 2C). Moreover, confocal microscopy demonstrated that a second PI3K inhibitor, wortmannin, abrogated the recruitment of the Btk-PH domain without interfering with bacteria binding to and clustering of CEACAM1 (Fig. 2D). Together, these results suggest that PI(3,4,5)P is locally produced by PI3Ks in response to CEACAM1 engagement and interference with PI3K activity impairs CEACAM1-mediated internalization of bacteria.

### CEACAM1-mediated endocytosis of meningococci by endothelial cells is also PI(3,4,5)P-dependent

**Figure 3:**
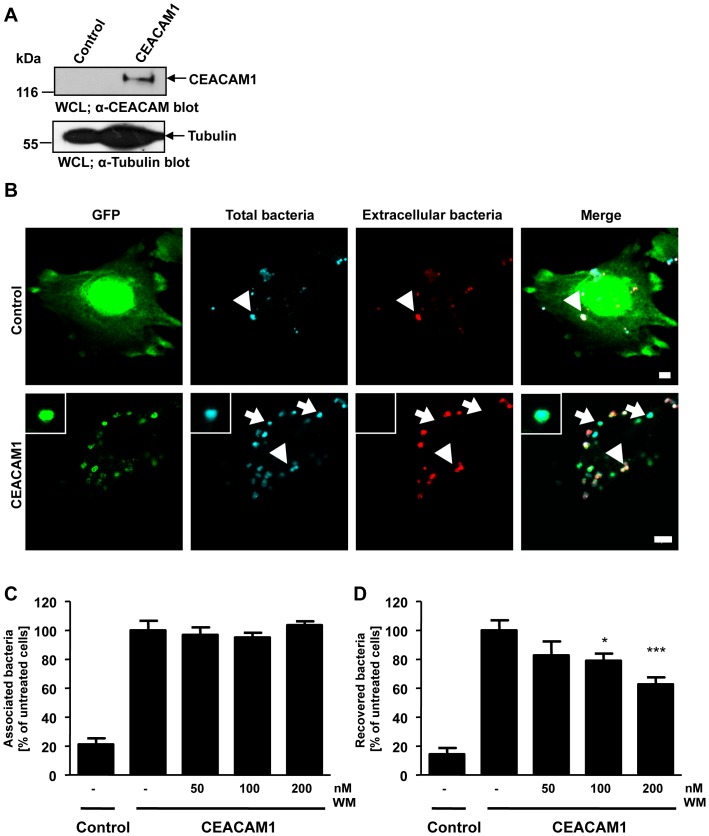
CEACAM1-mediated uptake of Opa_CEA_-expressing *N. meningitidis* by endothelial cells depends on PI3K activity. (**A**) Human brain microvascular endothelial cells (HBMEC) were transduced with GFP-encoding control lentivirus (control) or a CEACAM1-GFP-encoding lentivirus. CEACAM1 expression in transduced HBMEC is analysed by Western blotting of whole cell lysates (WCL) using monoclonal α-CEACAM antibody (upper panel) and equal loading of the samples was demonstrated by α- tubulin antibody (lower panel). (**B**) Stable CEACAM1-GFP or control GFP expressing HBMECs were infected with biotin- and AlexaFluor647-labelled Opa_CEA_-expressing meningococci for 60 min. Upon fixation and and staining of extracellular bacteria with streptavidin-rhodamine, samples were analyzed by confocal microscopy. Arrows highlight intracellular bacteria, whereas arrowheads point to extracellular bacteria. Bars represent 5 µm. (**C and D**) CEACAM1-GFP or GFP expressing HBMECs were pretreated with the indicated concentrations of wortmannin for 30 min or left untreated. Cells were infected for 3 h with Opa_CEA_-expressing meningococci and total cell-associated (C) or viable intracellular bacteria (D) were quantified. Bars represent mean values ± S.E.M of three independent experiments done in triplicate (n = 9). Numbers are expressed relative to CEACAM1-GFP cells without PI3K inhibitor treatment. Significance was tested using an unpaired, two-sided Student's t-test; ***, p<0.001, *, p<0.05.

CEACAM1 has also been shown to promote the internalization of *N. meningitidis* by endothelial cells [17]. To investigate, if the PI3K-dependence of CEACAM1-mediated bacterial internalization is a general feature in different cell types, we studied uptake of *N. meningitidis* by human brain-derived microvascular endothelial cells (HBMEC). Upon exposure to pro-inflammatory cytokines, endothelial cells are known to strongly upregulate CEACAM1 expression, which then allows internalization of Opa_CEA_-expressing meningococci [17], [42]. In a first step, we transduced HBMEC with recombinant lentivirus encoding CEACAM1-GFP or GFP alone to generate stable cell lines. Expression of CEACAM1-GFP in the transduced cells was verified by FACS analysis (data not shown) and Western Blotting (Fig. 3A). Next, HBMEC-GFP or HBMEC-CEACAM1-GFP were infected with Opa_CEA_-expressing *N. meningitidis* for 1 h and the fixed samples were differentially stained for intracellular and extracellular bacteria. Confocal microscopy revealed that small numbers of Opa_CEA_-expressing *N. meningitidis* adhered to GFP-expressing endothelial cells, but these bacteria were not internalized (Fig. 3B). In contrast, large numbers of meningococci were associated with CEACAM1-GFP expressed by HBMECs and a large proportion of the bacteria were internalized (Fig. 3B). Upon pre-treatment of CEACAM1-GFP expressing HBMECs with different concentrations of the PI3K inhibitor wortmannin, bacterial binding to the CEACAM1-GFP expressing endothelial cells was not affected (Fig. 3C). At the same time, the uptake of meningococci was decreased in a dose-dependent manner as measured by gentamicin protection assays (Fig. 3D) demonstrating that inhibition of PI(3,4,5)P generation affects CEACAM1-mediated endocytosis of bacteria in multiple cell types.

### Overexpression of constitutive active PI3K increases and expression of SHIP decreases uptake of Opa_CEA_-expressing gonococci via CEACAM1

**Figure 4:**
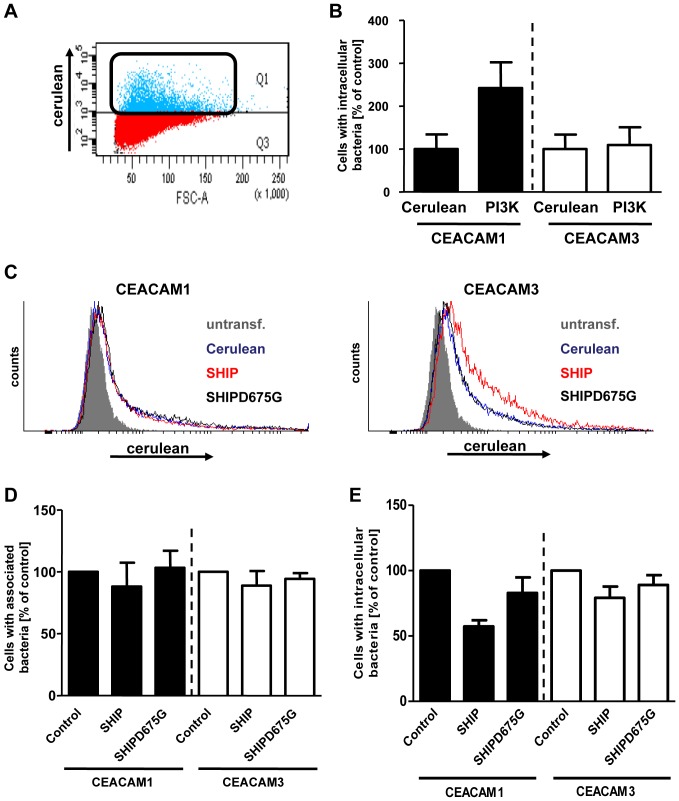
Constitutive active PI3K increases, whereas expression of SHIP decreases uptake of Opa_CEA_-expressing gonococci via CEACAM1. (**A**) 293 cells were cotransfected with constructs encoding CEACAM1-HA or CEACAM3-HA together with cerulean-tagged PI3K or cerulean alone. Cells were infected with fluorescein-labelled Opa_CEA_- expressing gonococci for 2 h. Samples were analysed using a flow cytometer by gating on cerulean-positive cells. Dot blot shows a representative gate used to detect the cerulean-positive cell population. (**B**) The fluorescein signal derived from cerulean-positive cells in (A) was quantified in the presence of trypan blue, which quenches fluorescence derived from extracellular gonococci. Bars represent mean values ± S.E.M of a representative experiment done in triplicate. (**C–E**) 293 cells were cotransfected with CEACAM1-HA or CEACAM3-HA together with cerulean, cerulean-tagged SHIP1 phosphatase domain (SHIP), or an inactive form of the SHIP1 phosphatase domain (SHIP D675G), respectively. (C) Expression of cerulean, SHIP1, or SHIP1 D675G was analysed by flow cytometry and histograms show representative samples. (D and E) Cells were infected with fluorescein-stained Opa_CEA_-expressing gonococci for 2 h and the fluorescein signal derived from cerulean-positive cells was detected in the absence (D) or presence (E) of trypan blue. This allows quantification of total cell-associated bacteria (D) or internalized gonococci (E). Bars represent mean values ± S.E.M of three independent experiments. Numbers are expressed relative to cells co-expressing cerulean and the respective receptor.

To further investigate the role of PI(3,4,5)P during CEACAM-mediated endocytosis, cells were cotransfected with vectors encoding CEACAM1 or CEACAM3 together with the cerulean-tagged enzymatic subunit (p110) of PI3K or cerulean alone, respectively. Cells were infected with fluorescein-labeled Opa_CEA_-expressing gonococci for 2 h and, after washing, the infected cells were analysed by flow cytometry by gating on the cerulean-positive, transfected cells (Fig. 4A). To specifically detect internalized bacteria, signals from cell-associated extracellular bacteria were quenched by the addition of trypan blue [38] and the fluorescein signal from internalized bacteria was recorded. Whereas CEACAM3-expressing cells did not show an altered uptake of bacteria upon overexpression of PI3K, CEACAM1-mediated uptake of Opa_CEA_-expressing gonococci was more than doubled in cells with elevated PI3K activity (Fig. 4B).

To further demonstrate that PI(3,4,5)P is critical for CEACAM1-mediated endocytosis, we overexpressed the PI(3,4,5)P-specific phosphatidylinositol-phosphatase SHIP, which dephosphorylates the 5′-position of PI(3,4,5)P to yield PI(3,4)P [43], [44]. The cerulean-tagged phosphatase domain of wildtype SHIP or an enzymatically inactive form of SHIP (SHIP D675G) were co-expressed with either HA-tagged CEACAM1 or HA-tagged CEACAM3, respectively. As a further control, cerulean alone was co-expressed with CEACAMs (control). Equivalent expression of cerulean-tagged SHIP constructs or cerulean was verified by flow cytometry (Fig. 4C). Next, cells were infected for 2 h with fluorescein-labeled Opa_CEA_-expressing gonococci and samples were analysed by gating on cerulean-positive cells during flow cytometry. Cell-association and internalization of fluorescein-labelled bacteria was measured in cerulean-positive cells in the absence (cell-association; Fig. 4D) or presence (invasion; Fig. 4E) of trypan blue, which quenches fluorescein fluorescence derived from extracellular bacteria. Overexpression of the wildtype SHIP phosphatase domain reduced uptake of bacteria via CEACAM1 by about 50%, whereas CEACAM3-mediated uptake was not affected (Fig. 4E). In contrast, overexpression of phosphatase-inactive SHIP D675G did not influence internalization via either of the receptors (Fig. 4E). Furthermore, binding of bacteria to CEACAM1- or CEACAM3-expressing cells was not altered upon co-expression of wildtype or phosphatase-inactive SHIP (Fig. 4D). Thus, modulation of cellular PI(3,4,5)P levels selectively affects CEACAM1-mediated internalization. The results with overexpression of PI3K or SHIP are in agreement with the idea that PI(3,4,5)P is critical for efficient CEACAM1-mediated endocytosis of bacteria.

### PI3K inhibition decreases bacterial uptake via CEACAM1 and CEACAM1ΔCT

**Figure 5:**
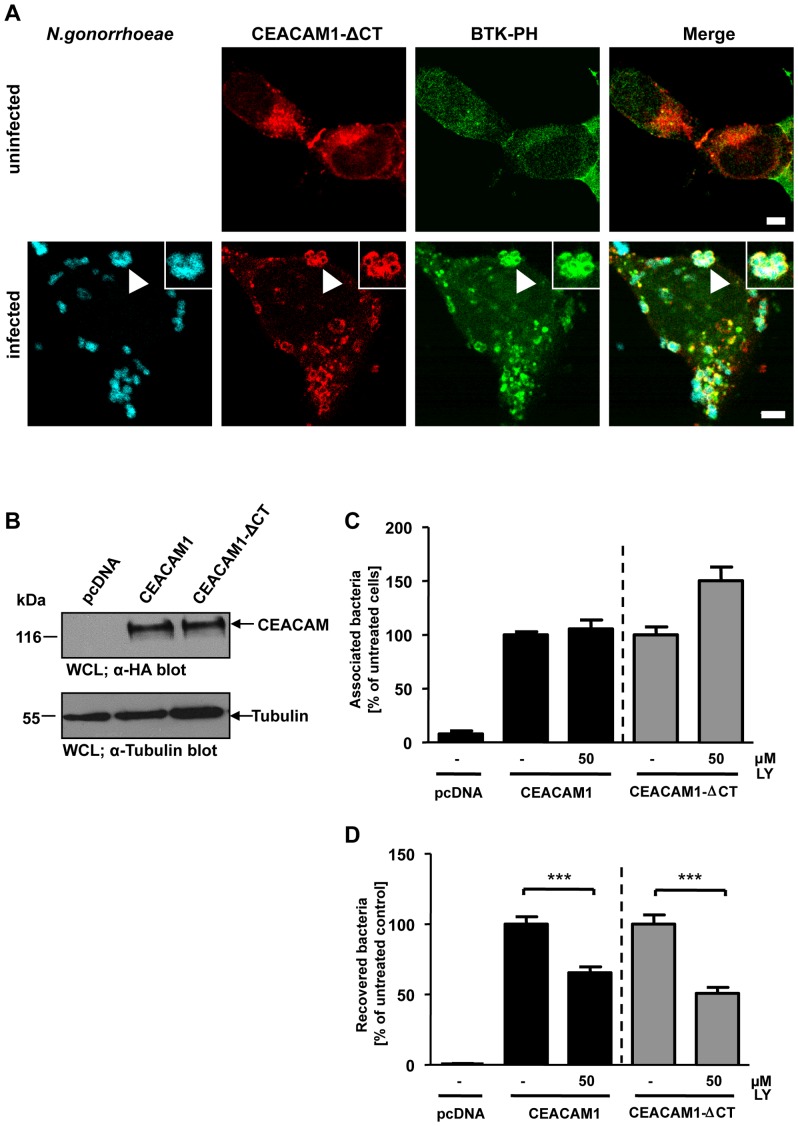
PI3K inhibition decreases bacterial uptake via CEACAM1 and CEACAM1-ΔCT. (**A**) 293 cells were co-transfected with mKate-tagged CEACAM1 lacking the cytoplasmic domain (CEACAM1-ΔCT) together with GFP-tagged Btk-PH domain and infected or not with Pacific Blue labelled Opa_CEA_-expressing gonococci for 60 min. After fixation, samples were analyzed by confocal microscopy. Arrowhead highlights CEACAM1-ΔCT clustering by gonococci and recruitment of Btk-PH domain. Insets show enlargement of the highlighted area. Bars represent 5 µm. (**B**) 293 cells were transfected with the empty vector (pcDNA), HA-tagged CEACAM1 wildtype or CEACAM1-ΔCT. CEACAM expression was analysed in whole cell lysates (WCLs) by Western blotting with α-HA antibody (upper panels) and equal loading of the samples was demonstrated by α-tubulin antibody (lower panel). (**C and D**) Cells transfected as in (B) were pretreated for 30 min with 50 µM of PI3K inhibitor LY294002 (LY) or left untreated. Cells were infected for 2 h with Opa_CEA_-expressing gonococci and total cell-associated (C) or viable intracellular bacteria (D) were quantified. Bars represent mean values ± S.E.M of three independent experiments done in triplicate (n = 9). Numbers are expressed relative to cells expressing the respective receptor without PI3K inhibitor treatment. Significance was tested using an unpaired, two-sided Student's t-test; ***, p<0.001.

The cytoplasmic domain of CEACAM1, and in particular the cytoplasmic domain of the long isoform of CEACAM1, can accommodate multiple protein-protein interactions [45]. Indeed, upon tyrosine phosphorylation, SH2-domain mediated binding of several proteins, including the regulatory subunit of class I PI3K, to the CEACAM1 cytoplasmic domain can be observed (Fig. S1A). Similarily, a direct association of PI3K with CEACAM3 has been reported and this interaction is mediated by the SH2 domains of PI3K binding to the cytoplasmic domain of CEACAM3 [31](Fig. S1B). However, a cytoplasmic domain is not required for efficient CEACAM1-mediated internalization of gonococci [28]. Therefore, we asked whether the cytoplasmic domain of CEACAM1 has a role in the local generation of PI(3,4,5)P. To this end, cells were co-transfected with constructs encoding the GFP-tagged Btk PH domain and mKate-tagged CEACAM1 lacking the cytoplasmic domain (CEACAM1 ΔCT). Upon infection with Pacific Blue-labelled CEACAM-binding *N. gonorrhoeae*, a strong recruitment of the Btk-PH domain was observed (Fig. 5A), demonstrating that PI(3,4,5)P generation in response to CEACAM1 engagement does not require the cytoplasmic domain of the receptor. To address the functional role of PI(3,4,5)P and of the CEACAM1 cytoplasmic domain, cells were transfected with constructs encoding HA-tagged CEACAM1 wildtype (CEACAM1), HA-tagged CEACAM1 ΔCT or the empty control vector (pcDNA). Similar expression levels of the receptors in the transfected cells were verified by Western blotting (Fig. 5B). The transfected cells were treated or not with the PI3K inhibitor wortmannin and infected with Opa_CEA_-expressing gonococci for 2 h. Whereas total numbers of cell-associated bacteria were not altered by PI3K inhibition (Fig. 5C), wortmannin severely decreased uptake of pathogens via both CEACAM1 and CEACAM1 ΔCT (Fig. 5D). These results suggest that the CEACAM1 cytoplasmic domain is not essential for PI(3,4,5)P generation required for CEACAM1-mediated uptake.

### Inhibition of PI3K activity does not affect CEACAM1 relocalisation to membrane microdomains

**Figure 6:**
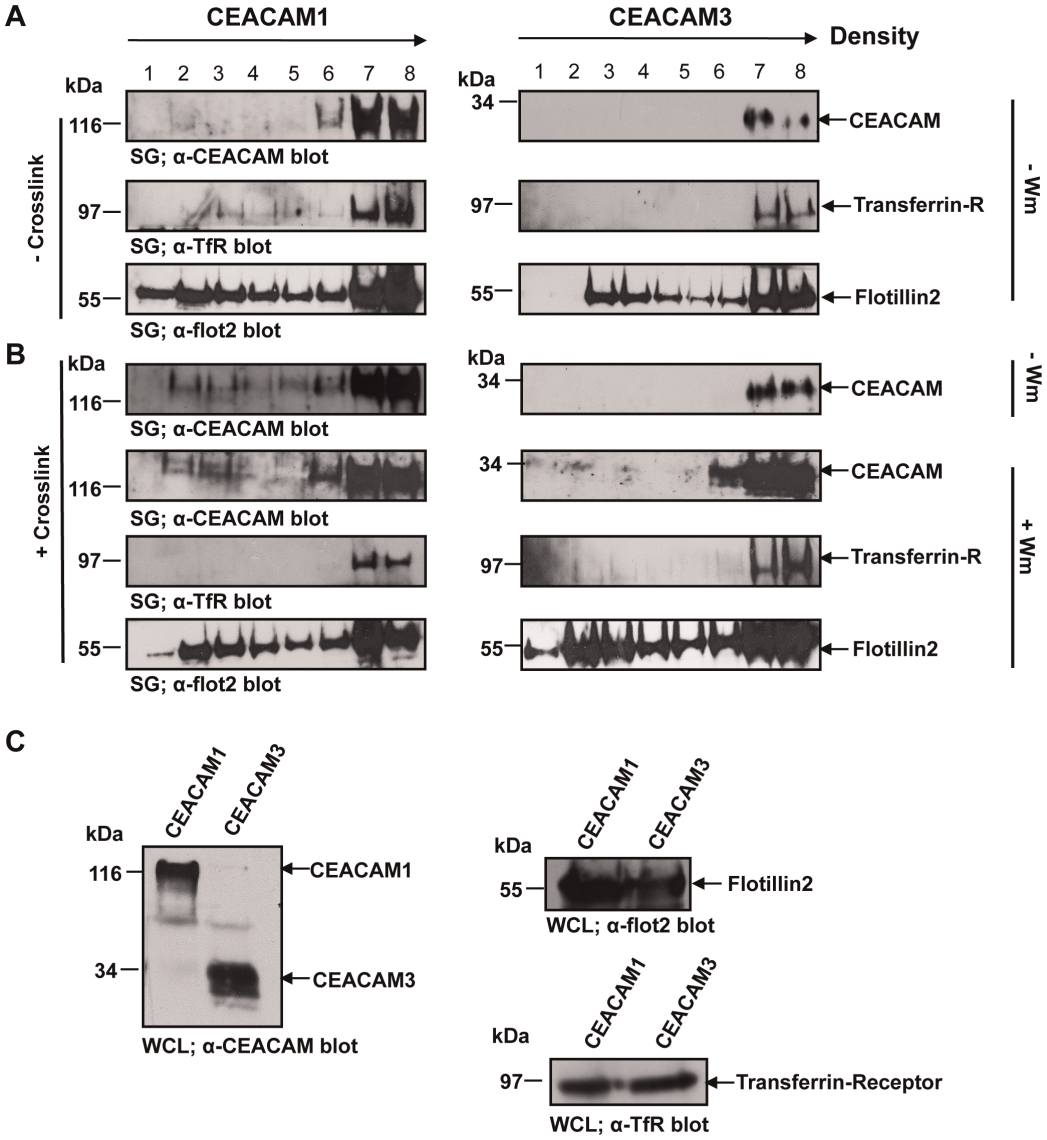
Inhibition of PI3K activity does not affect CEACAM1 relocalisation to membrane microdomains. (A) 293 cells were co-transfected with HA-tagged CEACAM1 or CEACAM3 together with GFP-tagged flotillin2. Following cells homogenization, the membrane fraction was extracted with 0.7% Triton X-100-containing buffer and loaded on a discontinous sucrose gradient (SG). After ultracentrifugation, eight distinct fractions were collected from top (low density: fraction 1) to bottom (high density: fraction 8) of the gradient and analyzed by Western blotting with antibodies against CEACAM, flotillin2 (flot2), or transferrin receptor (TfR). Detergent-resistant, low-density membrane components were identified mainly in fraction 2–4. (**B**) Cells were transfected as in (A) and CEACAMs were crosslinked using antibodies prior to homogenization. As indicated, some samples were treated for 30 min with 200 nM wortmannin (+WM) before crosslinking. Membrane fractions and Western blot analysis was as in (A). (**C**) Expression of CEACAMs, TfR, or flot2 in whole cell lysates (WCLs) was determined by Western blotting with the indicated antibodies.

A main difference between CEACAM1- and CEACAM3-mediated endocytosis concerns the membrane distribution of these receptors. Whereas CEACAM1 has been shown to translocate into membrane microdomains (lipid rafts) after receptor engagement, CEACAM3 remains in the non-raft membrane fraction [28]. Interestingly, membrane microdomain association of CEACAM1 occurs also in the absence of a cytoplasmic domain [28]. To investigate, whether the abundance of PI(3,4,5)P affects the relocalization of CEACAM1 into membrane microdomains, we analysed detergent-resistant membrane fractions of CEACAM-expressing cells in the presence or absence of a PI3K inhibitor. Before receptor clustering, CEACAM1 and CEACAM3 were found in a high-density membrane fraction similar to the non-lipid raft-associated transferrin receptor (TfR) (Fig. 6A). As observed before, receptor-clustering promoted a re-distribution of CEACAM1, but not CEACAM3, into the low-density membrane fractions, which were also enriched for the lipid raft marker protein flotillin2 (Fig. 6B). Importantly, pre-treatment of CEACAM1-expressing cells for 30 min with 200 nM wortmannin did not prohibit the re-distribution of CEACAM1 into membrane microdomains upon crosslinking (Fig. 6B). Similar expression of CEACAM1 and CEACAM3, as well as the membrane marker proteins TfR and flotillin2 in the employed cell lysates was verified by Western Blotting (Fig. 6C). These results demonstrated that membrane microdomain localization of CEACAM1 is not influenced by the generation of PI(3,4,5)P and suggested that CEACAM1-initiated PI3K activity could be downstream of CEACAM1 re-distribution to lipid rafts.

### Membrane microdomain localization is not required for CEACAM1-initiated PI(3,4,5)P generation

**Figure 7:**
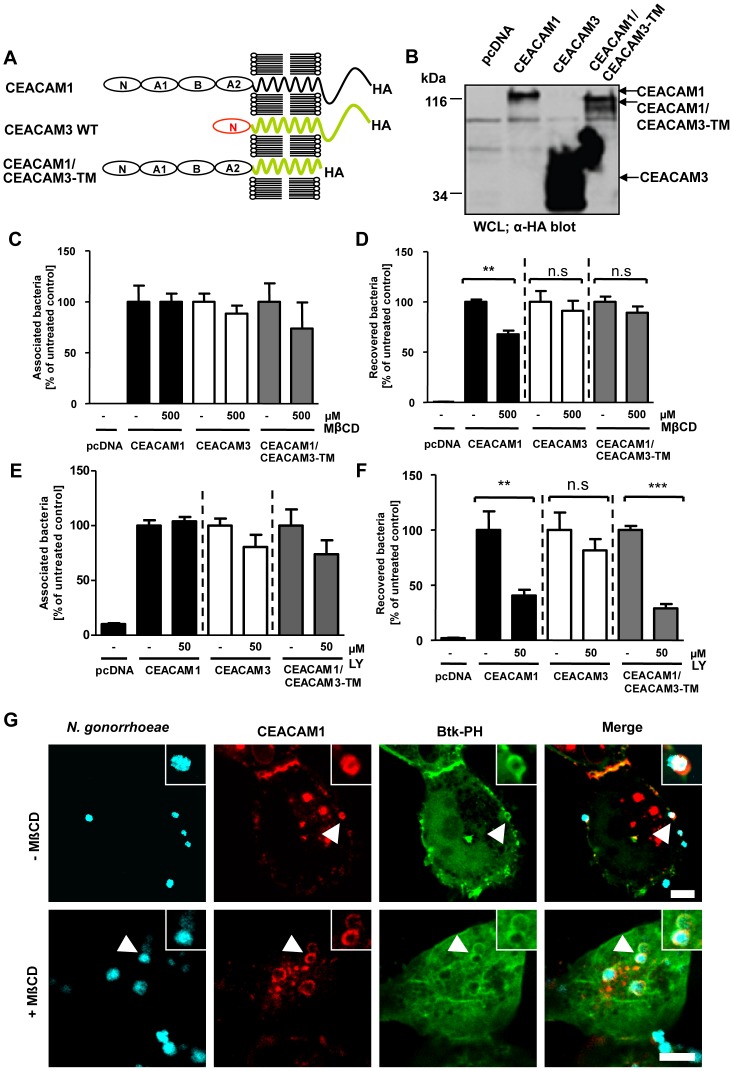
Membrane microdomain localization is not required for CEACAM1-initiated PI(3,4,5)P generation. (**A**) Domain organization of the used CEACAM constructs. N – Ig_V_-like N-terminal domain; A1, B, A2 – Ig_C2_-like domains. (**B**) 293 cells were transfected with empty vector (pcDNA) or constructs encoding HA-tagged CEACAM1, CEACAM3, or a chimeric protein consisting of the extracellular domains of CEACAM1 fused to the transmembrane domain of CEACAM3 (CEACAM1/CEACAM3-TM). Expression was verified by Western blotting with α-HA antibody. (**C, D**) Cells transfected as in (B) were pretreated for 30 min with 500 µM methyl-β-cyclodextrin (MβCD) or left untreated. After infection for 2 h with Opa_CEA_-expressing gonococci total cell-associated bacteria (C) or recovered intracellular bacteria (D) were quantified. Bars represent mean ± SEM of three independent experiments done in triplicate (n = 9). Numbers are expressed relative to cells expressing the respective receptor without MβCD treatment. Significance was tested using an unpaired, two-sided Student's t-test; **, p<0.01; n.s. – not significant. (**E, F**) Cells transfected as in (B) were pretreated with 50 µM of LY294002 (LY). Cells were infected as in (C, D) and total cell-associated bacteria (E) or recovered intracellular bacteria (F) were quantified. Bars represent mean ± SEM of three independent experiments done in triplicate (n = 9). Numbers are expressed relative to cells expressing the respective receptor without LY treatment. Significance was tested using an unpaired, two-sided Student's t-test; ***, p<0.001; **, p<0.01; n.s. – not significant. (**G**) 293 cells were transfected with mKate-tagged CEACAM1 together with GFP-tagged Btk-PH domain and treated with MβCD before infection with Pacific Blue labelled Opa_CEA_-expressing gonococci. After 60 min, samples were fixed and analyzed via confocal microscopy. Arrowheads highlight gonococci associated with clustered CEACAM1. Insets show enlargement of the highlighted area. Bars represent 5 µm.

To test the idea, that membrane microdomain localization might be a pre-requisite for the CEACAM1-dependent generation of PI(3,4,5)P, we employed a chimeric receptor, CEACAM1/CEACAM3-TM, which encompasses the extracellular domain of CEACAM1 fused to the transmembrane domain (TM) of CEACAM3 (Fig. 7A). Expression of CEACAM1, CEACAM3 and the chimeric receptor was verified by Western Blotting (Fig. 7B). Previously, we have shown that the TM of CEACAM1 directs the receptor into membrane microdomains, whereas the CEACAM3 TM prohibits lipid raft localization [28]. Involvement of lipid rafts can be revealed by methyl-β-cyclodextrin (MβCD), a cholesterol chelator, which removes cholesterol from cellular membranes, thereby disrupting lipid raft integrity and interfering with CEACAM1-mediated uptake of gonococci [22], [28]. As expected and similar to CEACAM3, bacterial endocytosis via CEACAM1/CEACAM3-TM was insensitive to methyl-β-cyclodextrin (MβCD) treatment (Fig. 7D), whereas total numbers of cell-associated bacteria were not altered (Fig. 7C). Nevertheless, bacterial internalization via CEACAM1/CEACAM3-TM was still sensitive to PI3K inhibition by LY294002 (Fig. 7F) suggesting that this lipid raft-independent uptake still involved PI(3,4,5). To confirm that membrane microdomain localization of CEACAM1 is not directly connected to CEACAM1-dependent PI(3,4,5)P generation, cells expressing CEACAM1-mKate together with the GFP-tagged BTK-PH domain were treated or not with MβCD. Confocal microscopy confirmed that the Btk-PH domain was recruited to the site of bacterial CEACAM1 engagement (Fig. 7G). Interestingly, cholesterol depletion by MβCD only slightly reduced the local accumulation of PI(3,4,5)P (Fig. 7G). Therefore, the integrity of lipid rafts is not essential for PI3K activation in response to CEACAM1 stimulation. Together, these results suggest that redistribution into membrane microdomains and the receptor-initiated generation of PI(3,4,5) are two independent events needed for optimal CEACAM1-mediated endocytosis.

### Bacterial uptake via several epithelial CEACAMs requires PI3K activity

**Figure 8:**
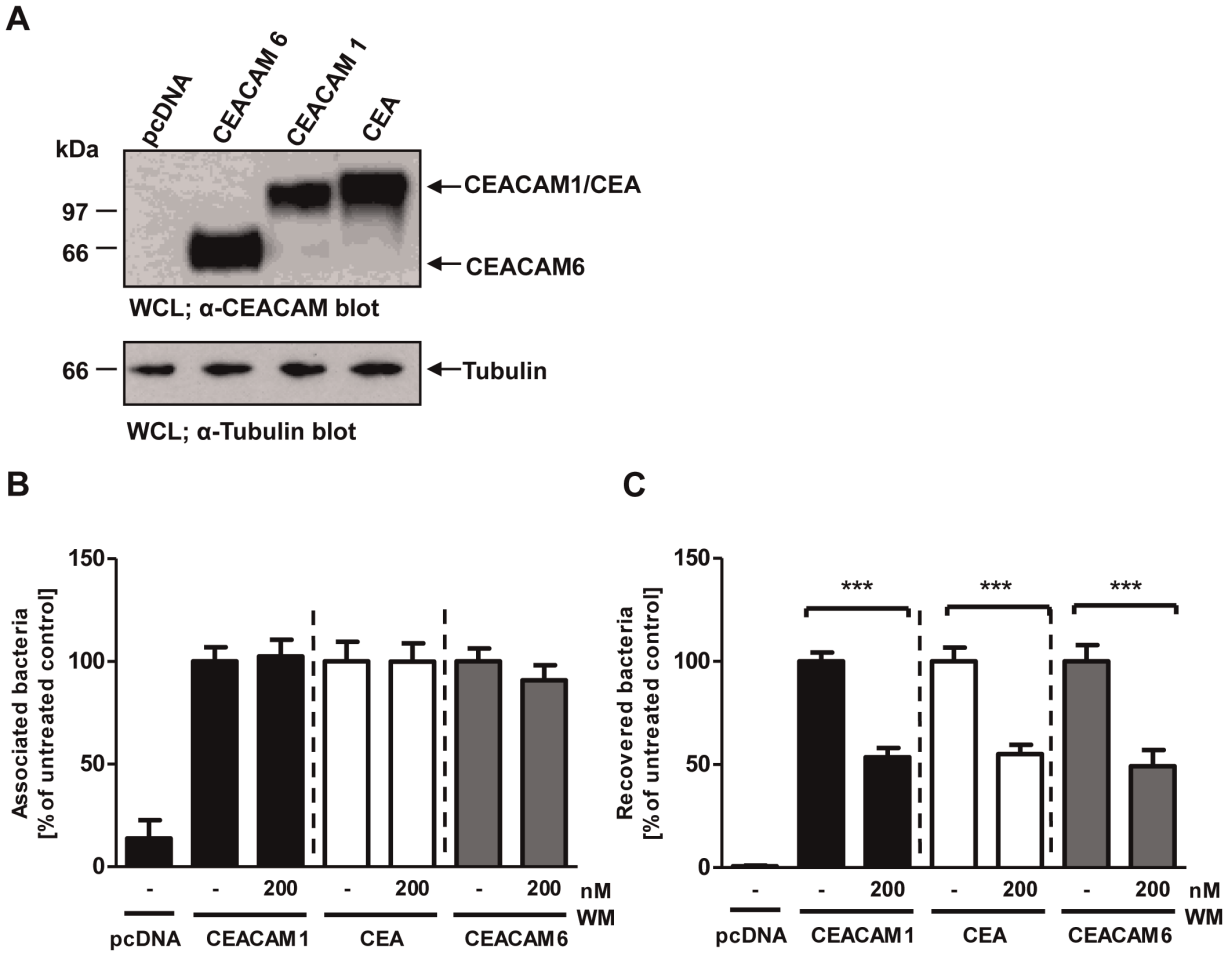
Bacterial uptake via several epithelial CEACAMs requires PI3K activity. (**A**) 293 cells were transfected with empty vector (pcDNA) or constructs encoding CEACAM1, CEA, or CEACAM6. CEACAM expression was confirmed by Western blotting of whole cell lysates (WCLs) with α-CEACAM antibody (upper panel). Equal loading of the samples was verified by α-tubulin blot (lower panel). (**B, C**) Cells transfected as in (A) were pretreated for 30 min with 200 nM wortmannin (WM) or left untreated. After infection for 2 h with Opa_CEA_-expressing gonococci, total cell-associated bacteria (B) or recovered intracellular bacteria (C) were quantified. Bars represent mean ± SEM of three independent experiments done in triplicate (n = 9). Numbers are expressed relative to cells expressing the respective receptor without WM treatment. Significance was tested using an unpaired, two-sided Student's t-test; ***, p<0.001.

Neither the cytoplasmic part of CEACAM1 nor the transmembrane domain-mediated recruitment to membrane microdomains were mechanistically linked to the PI3K-dependent endocytosis of bacteria. Therefore, we wondered, whether bacterial uptake via GPI-linked epithelial CEACAMs, which lack cytoplasmic and transmembrane domains, also requires the generation of PI(3,4,5)P. Accordingly, cells were transfected with constructs encoding HA-tagged CEACAM1, CEA, CEACAM6, or the empty control vector (pcDNA). Expression levels were confirmed by Western blot with a monoclonal antibody recognizing several human CEACAMs (Fig. 8A). Next, cells were infected for 2 h with Opa_CEA_-expressing gonococci in the presence or absence of the PI3K inhibitor wortmannin and adherence of the bacteria to the cells as well as internalization of the bacteria were quantified (Fig. 8B and C). As observed for CEACAM1, a significant reduction of bacterial internalization was seen for CEA and CEACAM6 upon addition of the PI3K inhibitor, whereas the total amount of cell-associated bacteria was not affected (Fig. 8B and C). These results point to a general requirement for PI3K activity and PI(3,4,5)P during endocytosis of bacteria via epithelial CEACAMs.

### Extracellular Ig_C2_-like domains of CEACAM1 control the PI3K dependency during pathogen uptake

**Figure 9:**
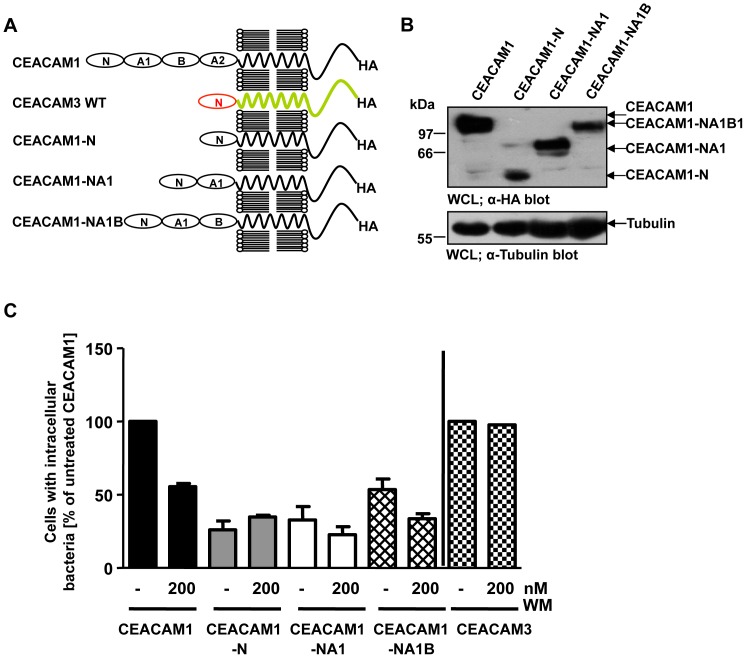
Extracellular Ig_C2_-like domains of CEACAM1 are required for the PI3K dependency during pathogen uptake. (**A**) Domain organization of the used CEACAM constructs. N – Ig_V_-like N-terminal domain; A1, B, A2 – Ig_C2_-like domains. (**B**) 293 cells were transfected with HA-tagged CEACAM1, CEACAM1-N, CEACAM1NA1 or CEACAM1NA1B. CEACAM expression was confirmed by Western blotting with α-HA antibody (upper panel). Equal loading of the samples was verified by α-tubulin blot (lower panel). (**C**) Cells transfected as in (B) were pretreated for 30 min with 200 nM wortmannin (WM) or left untreated before infection with fluorescein-labelled Opa_CEA_-expressing gonococci for 2 h. Infected cells were analysed by flow cytometry and the fluorescein signal from internalized bacteria was detected in the presence of trypan blue, which quenches fluorescence derived from extracellular gonococci. Bars represent mean values ± S.E.M from three independent experiments. Numbers are expressed relative to cells expressing CEACAM1 without WM treatment.

Besides the localization in membrane microdomains, CEA and CEACAM6 share with CEACAM1 the presence of additional extracellular Ig_C2_ domains. Interestingly, CEACAM3 does not encompass Ig_C2_ domains and has only the single N-terminal Ig_V_-like domain, which is characteristic of CEACAM family members. As both the transmembrane and the cytoplasmic domain of CEACAM1 were not involved in the PI3K-dependent uptake of bacteria, we asked, whether the extracellular Ig_C2_ domains of CEACAM1 could be a critical determinant for this process. Therefore, we generated different deletion constructs of CEACAM1 lacking either one (CEACAM1-NA1B), two (CEACAM1-NA1), or all (CEACAM1-N) extracellular Ig_C2_-like domains (Fig. 9A). Cells were transfected with the different constructs and all proteins were expressed at similar levels except for CEACAM1-N, which was expressed at slightly lower levels (Fig. 9B). The transfected cells were treated or not with PI3K inhibitor prior to infection with fluorescein-labelled Opa_CEA_-expressing gonococci. 2 h after the infection, cells were analysed by flow cytometry for intracellular bacteria. As before, PI3K inhibition reduced internalization of bacteria via wildtype CEACAM1, but also CEACAM1-NA1B and CEACAM1-NA1 showed decreased uptake of Opa_CEA_-expressing *N. gonorrhoeae* upon PI3K inhibition (Fig. 9C). Significantly, for CEACAM1-N, which lacks all extracellular Ig_C2_ domains and which supports only low level of internalization, no further reduction of bacterial uptake upon PI3K inhibition was observed compared to the uptake in the absence of the inhibitor (Fig. 9C). Clearly, the absolute number of endocytosed bacteria was lower for CEACAM1 deletion mutants lacking one or more Ig_C2_ domains compared to wildtype CEACAM1. Together with the marginal or absent effect of wortmannin on uptake via the CEACAM1 deletion mutants, these results suggest that the Ig_C2_-like domains are responsible for connecting CEACAM1 to a PI3K-dependent endocytosis pathway.

### Overexpression of a CEACAM8/1 chimera interferes with CEACAM1-mediated uptake

**Figure 10:**
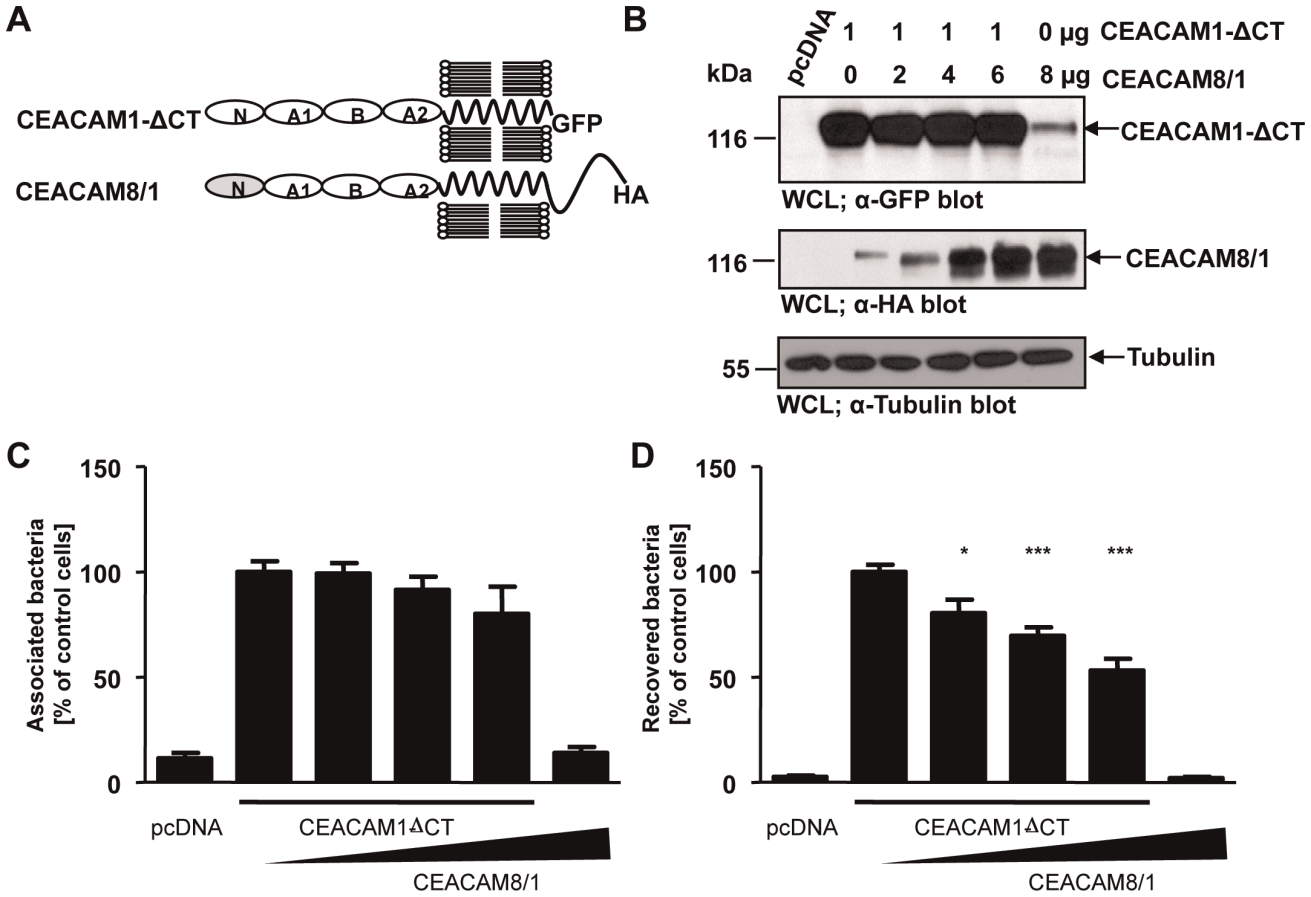
Overexpression of a CEACAM8/1 chimera interferes with CEACAM1-mediated uptake. (**A**) Domain organization of the used CEACAM constructs. N – Ig_V_-like N-terminal domain; A1, B, A2 – Ig_C2_-like domains. (**B**) 293 cells were transfected with empty vector (pcDNA) or a construct encoding CEACAM1 ΔCT-GFP (1 µg) together with increasing amounts of a plasmid encoding the HA-tagged CEACAM8/1 chimera (0 to 6 µg). One sample was transfected with CEACAM8/1-HA (6 µg) only. Differential expression of CEACAM1 ΔCT-GFP and CEACAM8/1-HA was verified by Western blotting of whole cell lysates (WCLs) using α-GFP antibody (CEACAM1 ΔCT; upper panel) or α-HA antibody (CEACAM8/1; middle panel), respectively. Equal loading of samples was demonstrated by Western blotting with α-tubulin antibody (lower panel). (**C, D**) Cells transfected as in (B) were infected with Opa_CEA_-expressing gonococci. After infection for 2 h, total cell-associated bacteria (C) or recovered intracellular bacteria (D) were quantified. Numbers are expressed relative to cells expressing CEACAM1 ΔCT in the absence of CEACAM8/1. Bars represent mean ± SEM of three independent experiments done in triplicate (n = 9). Significance was tested using an unpaired, two-sided Student's t-test; ***, p<0.001; *, p<0.05.

If the Ig_C2_-like domains connect epithelial CEACAMs to additional membrane protein(s), then overexpression of Ig_C2_ domains in the absence of a bacteria-binding Ig_V_-like domain should block CEACAM1-mediated internalization. To test this idea, we took advantage of CEACAM8, a member of the CEACAM family, which is not recognized by Opa_CEA_-expressing gonococci [22], [46]. The CEACAM8 N-terminal Ig_V_-like domain was fused to the Ig_C2_-like domains of CEACAM1 creating a CEACAM8/1 chimera (Fig. 10A). Next, we co-transfected increasing amounts of the construct encoding the HA-tagged CEACAM8/1 chimera together with a constant amount of GFP-tagged CEACAM1 lacking the cytoplasmic domain (CEACAM1 ΔCT) and verified the expression in whole cell lysates by Western blotting (Fig. 10B). Control cells were transfected with the empty vector (pcDNA) or with the CEACAM8/1 chimera alone (Fig. 10B). Importantly, increasing amounts of CEACAM8/1 chimera on the cell surface did not alter the total amount of cell-associated bacteria (Fig. 10C). However, the increased presence of CEACAM8/1 resulted in a corresponding decrease in bacterial internalization (Fig. 10D). These results support the idea that the Ig_C2_ domains of CEACAM1 are involved in the uptake of bacteria, most likely via lateral interaction with other cell surface receptor(s). Together, our data point to a CEACAM co-receptor, which might be able to associate with epithelial CEACAMs via their Ig_C2_-like domains and which appears to regulate endocytosis in a PI3K- and PI(3,4,5)P-dependent manner.

## Discussion

CEACAM family members on epithelial cells are the target of several human restricted bacteria, which exploit these receptors for host cell attachment and internalization [2]. Here we demonstrate that endocytosis of bacteria via the epithelial CEACAM family members CEACAM1, CEA, and CEACAM6 requires PI3 kinase activity. Pharmacological inhibition of PI3 kinase enzyme activity or overexpression of a PI(3,4,5)P-specific phosphatase inhibit CEACAM1-mediated uptake, whereas overexpression of class I PI3-kinase augments the internalization of bacteria via CEACAM1. PI3-kinase activity was not connected to the specific subcellular localization of CEACAM1 in cholesterol-rich membrane microdomains, but required the presence of extracellular immunoglobulin domains of CEACAM1 suggesting that CEACAM1 engages in a cis-interaction with other membrane proteins to trigger endocytosis.

In contrast to CEACAM1, PI3K inhibition by wortmannin does not affect CEACAM3-mediated uptake of bacteria by transfected cell lines or primary human granulocytes [31]. Bacterial internalization via CEACAM3 has been studied in great detail and depends on actin cytoskeleton dynamics orchestrated by sequence determinants in the CEACAM3 cytoplasmic domain [21]. Indeed, CEACAM3 engagement results in massive lamellipodial protrusions on the cell surface tightly connected to bacterial engulfment [24], [27], [47]. Therefore, the independence of CEACAM3-mediated uptake from PI3K activity is particularly intriguing, as PI(3,4,5)P has been linked to the local regulation of actin polymerization and membrane trafficking via recruitment and activation of guanine nucleotide exchange factors (GEFs) for small GTPases of the Rho and Arf families [48]. However, phosphorylated CEACAM3 can directly associate with Vav to promote GTP loading of the small G-protein Rac and this direct binding to a Rac GEF might allow CEACAM3 to bypass a requirement for PI3K activity during phagocytosis [21], [26]. On the other hand, actin cytoskeleton dynamics play only a minor role during uptake via epithelial CEACAMs, which occur with a slower kinetic and in the absence of major surface protrusions compared to CEACAM3-mediated uptake [22], [24], [30]. Therefore, PI(3,4,5)P generation in response to CEACAM1 engagement does not seem to be necessary for driving prominent actin-based changes at the cell surface. Previous studies have indicated that PI(4,5)P, which is the substrate of class I PI3K and is the major phosphoinositide synthesized at the plasma membrane, has to be removed from invaginated membranes to allow endosome formation via dynamin binding [49], [50]. For example, invasion of Yersinia into epithelial cells can only proceed from a semi-enclosed state to a fully-enclosed state upon removal of PI(4,5)P [51]. Though there are multiple ways to locally reduce PI(4,5)P levels, recruitment and activation of class I PI3K, which utilizes PI(4,5)P to generate PI(3,4,5)P, is one possible route to diminish PI(4,5)P to allow completion of CEACAM1-mediated endocytosis. However, the fact that overexpression of SHIP, which degrades PI(3,4,5)P to PI(3,4)P, has a negative impact on CEACAM1-mediated internalization argues against the idea that PI3K function in this process is instrumental for reducing PI(4,5)P levels at the plasma membrane.

Another characteristic difference between epithelial CEACAMs and CEACAM3 relates to the distinct distribution in membrane microdomains [22], [28]. As a result, bacterial uptake via epithelial CEACAMs is sensitive to cholesterol chelators such as methyl-β-cyclodextrin. However, MβCD treatment did not abolish the generation of PI(3,4,5)P at sites of bacterial-CEACAM1 contact and PI3K inhibition did not alter the distribution of CEACAM1 in membrane microdomains. These results suggest that two independent pre-requisites, membrane microdomain localization and PI3K activation, have to occur together to allow optimal CEACAM1 endocytosis. Most importantly, our results imply that these pre-requisites are coordinated by different molecular determinants of epithelial CEACAMs. On the one hand, the GPI anchor of CEA as well as CEACAM6 or the transmembrane domain of CEACAM1 direct these receptors into membrane microdomains [28]. On the other hand, the Ig_C2_-like extracellular domains, presumably by associating with a so far unknown co-receptor, are responsible for connecting CEACAM1 with PI3K activity. It is currently unknown if the Ig_C2_ domains of all epithelial CEACAMs connect to the same co-receptor. Sequence comparisons of Ig_C2_ domains of CEACAM1, CEA, and CEACAM6 reveal that these domains share 90–95% similarity between these proteins, which is comparable to the high similarity found for the N-terminal Ig_V_-like domains. This degree of conservation of the Ig_C2_ domains could allow these proteins to interact laterally with the same kind of co-receptor. However, further investigations depend on the identification of such a co-receptor.

One hint on the identity of a potential co-receptor might come from studies of other pathogenic bacteria, which invade epithelial cells in a PI(3,4,5)P-dependent manner. For example, *Listeria monocytogenes* triggers a PI3K-dependent uptake pathway by binding to the receptor tyrosine kinase (RTK) c-Met [52], [53]. RTKs such as c-Met might be perfect co-receptors for epithelial CEACAMs. Indeed, several RTKs possess, in addition to their ligand binding regions, extracellular protein-protein-interaction domains including Ig domains, fibronectin type III repeats, cadherin or discoidin domains [54]. Previously, a functional interaction between rat CEACAM1 and the insulin receptor (IR) has been reported that effects endocytosis of the IR together with its insulin ligand [55], [56]. At the same time, insulin triggers endocytosis of CEACAM1 in hepatocytes, presumably in a complex together with the insulin-bound IR [57]. However, the IR-initiated endocytosis of CEACAM1 is regulated by amino acid residues located in the long cytoplasmic domain of CEACAM1 [57], [58] making it unlikely that the IR is responsible for PI3K-dependency of endocytosis of epithelial CEACAMs.

Another example of a pathogen invading host cells in a PI3K-dependent manner is provided by *E. coli* K1, which causes meningitis in neonates. Invasion of these bacteria into brain microvascular endothelial cells is blocked by pharmacological PI3K inhibition or overexpression of dominant-negative variants of class I PI3Ks [59]. Host cell receptors for the *E. coli* K1 OmpA protein on endothelial and epithelial cells have been identified as Ecgp and gp96, respectively [60]. Gp96 (also known as GRP94 or HSP90b1) is a glycoprotein of the Hsp70 chaperone family and located in the ER lumen, but small amounts are also found on the cell surface [61]. Gp96 interacts with numerous membrane proteins such as integrins and Toll-like receptors [62]. How *E. coli* K1 OmpA-mediated binding to gp96 triggers PI3K-dependent internalization is currently unclear. As gp96 is linked to multiple eukaryotic surface receptors, it might be a candidate protein involved in the PI(3,4,5)P-dependent uptake of bacteria via CEACAM1 and other epithelial CEACAMs.

*Streptococcus pneumoniae* is a further pathogen, which invades epithelial cells in a PI3K-dependent manner [63]. In this context, the *S. pneumoniae* protein PspC engages immunoglobulin-like extracellular domains of the polymeric immunoglobulin receptor (pIgR) to trigger uptake into respiratory epithelial cells [64]. Interestingly, the physiological function of pIgR is to regulate the transcytosis of IgA and IgM from the basolateral membrane to the apical side of mucosal epithelia. Upon arrival at the apical surface, the pIgR is proteolytically processed. Whereas the extracellular domain, as so-called secretory component, is released together with its cargo antibody, the truncated pIgR can be endocytosed from the apical membrane [65]. Moreover, a minor fraction of intact pIgR seems to undergo transcytosis from the apical surface to the basolateral membrane [65]. As epithelial CEACAMs have been reported to allow transcytosis of *N. gonorrhoeae* across polarized epithelia, the pIgR and its PI(3,4,5)P-dependent endocytosis appear as promising candidates for a cis-interacting membrane protein. However, pIgR is only expressed on mucosal epithelia and not found on endothelial cells and therefore, could not be the responsible for the PI3K-dependent endocytosis of CEACAM1 by endothelial cells.

Together, our studies point to the existence of co-receptor(s) for epithelial CEACAMs, which confer specific signalling properties to these bacterial target molecules. Surprisingly, protein-protein interactions mediated by the Ig_C2_ domains of epithelial CEACAMs appear to be responsible for the PI3K-dependent endocytosis. Therefore, our novel results explain the ability of CEACAM1, CEA, and CEACAM6 to mediate bacterial uptake in the absence of cytoplasmic domains. Besides the connection to PI3K activation and the presence of extracellular protein-protein interaction domains, a putative CEACAM co-receptor should be expressed by epithelial and endothelial cells. Clearly, identification of a co-receptor for epithelial CEACAMs should be the next step to understand the role of epithelial CEACAMs as pathogen receptors and potentially obtain new insight into their physiological functions.

## Supporting Information

Figure S1 **The amino-terminal SH2 domain of the regulatory subunit of class I PI3K associates with phosphorylated CEACAM1 and CEACAM3.** 293 cells were transfected with HA-tagged CEACAM1 or GFP-tagged CEACAM3 constructs together with or without v-Src, a constitutive active protein tyrosine kinase. (A) CEACAM1-HA expressing cells were lysed and pulldown assays (PD) were performed with GST alone, GST-tagged SHP2-C-SH2 or GST-tagged PI3K-N-SH2 domain immobilized on glutathione-sepharose beads. Following washing, precipitates were analyzed for the presence of CEACAM1 by Western blotting with α-HA antibody. Both SHP2-C-SH2 and PI3K-N-SH2 were able to precipitate CEACAM1 from the lysate, whereas CEACAM1 was not found in precipitates of GST alone (upper panels). The presence of equivalent amounts of GST fusion proteins in the precipitates was demonstrated via Coomassie staining of the membrane (lower panels). CEACAM1 expression and tyrosine phosphorylation were confirmed by Western blotting with α-HA and α-pTyr antibodies (right panels). (B) CEACAM3 expressing cells were lysed and pulldown assays (PD) were performed with GST alone, GST-tagged Hck-SH2 or GST-tagged PI3K-N-SH2 domain. CEACAM3 expression and phosphorylation as well as its association with SH2 domains was analysed as in (A). The results verified that phosphorylated CEACAM3 bound to the SH2 domains of Hck and PI3K as previously reported.
(TIF)
