# Supplementary figures and images for: Extracellular IgC2 Constant Domains of CEACAMs Mediate PI3K Sensitivity during Uptake of Pathogens
Source: PLoS One. 2012 Jun 29;7(6):e39908. doi: 10.1371/journal.pone.0039908 (PMC3386982; doi:10.1371/journal.pone.0039908)

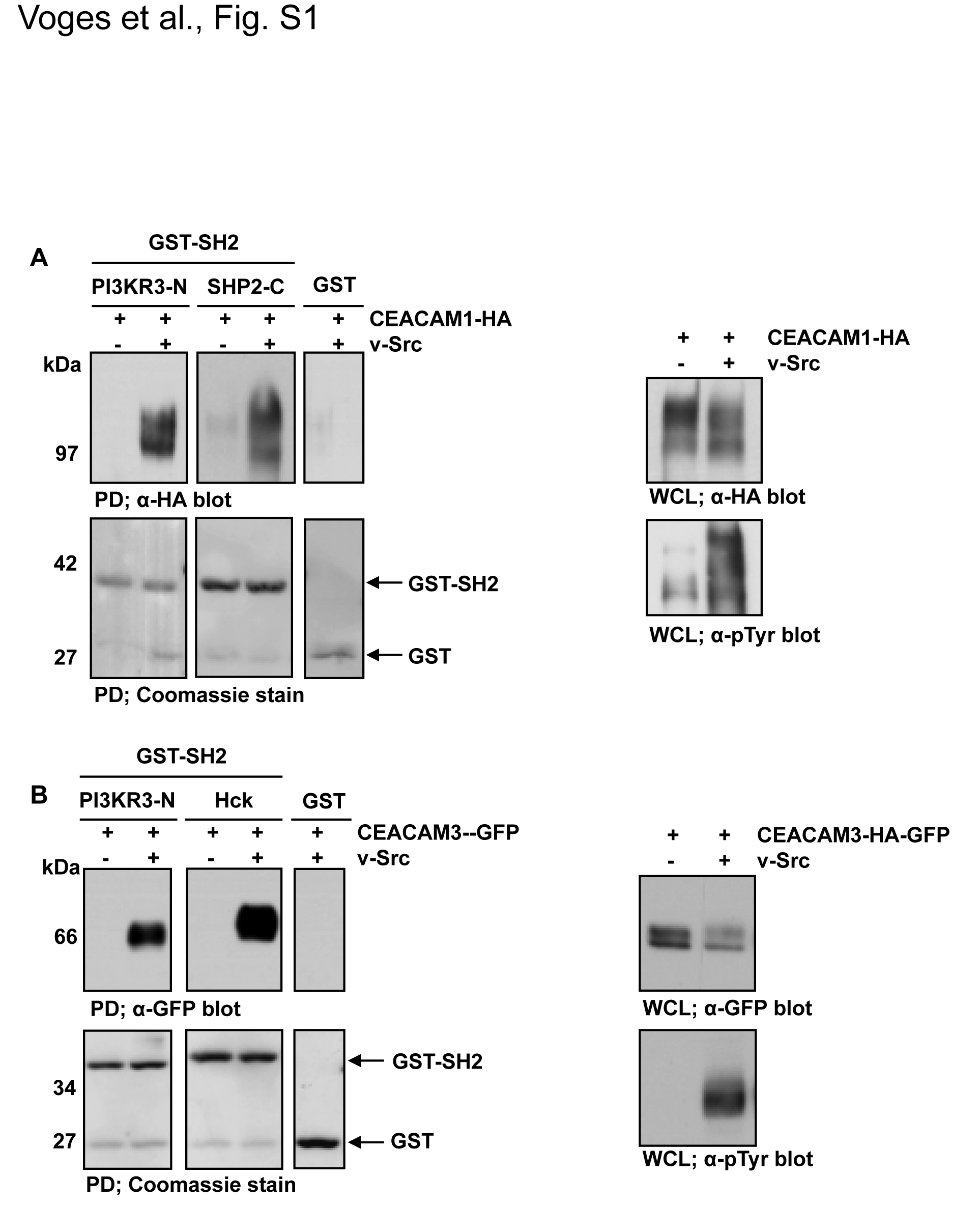

Supplement: Figure S1 — The amino-terminal SH2 domain of the regulatory subunit of class I PI3K associates with phosphorylated CEACAM1 and CEACAM3. 293 cells were transfected with HA-tagged CEACAM1 or GFP-tagged CEACAM3 constructs together with or without v-Src, a constitutive active protein tyrosine kinase. (A) CEACAM1-HA expressing cells were lysed and pulldown assays (PD) were performed with GST alone, GST-tagged SHP2-C-SH2 or GST-tagged PI3K-N-SH2 domain immobilized on glutathione-sepharose beads. Following washing, precipitates were analyzed for the presence of CEACAM1 by Western blotting with α-HA antibody. Both SHP2-C-SH2 and PI3K-N-SH2 were able to precipitate CEACAM1 from the lysate, whereas CEACAM1 was not found in precipitates of GST alone (upper panels). The presence of equivalent amounts of GST fusion proteins in the precipitates was demonstrated via Coomassie staining of the membrane (lower panels). CEACAM1 expression and tyrosine phosphorylation were confirmed by Western blotting with α-HA and α-pTyr antibodies (right panels). (B) CEACAM3 expressing cells were lysed and pulldown assays (PD) were performed with GST alone, GST-tagged Hck-SH2 or GST-tagged PI3K-N-SH2 domain. CEACAM3 expression and phosphorylation as well as its association with SH2 domains was analysed as in (A). The results verified that phosphorylated CEACAM3 bound to the SH2 domains of Hck and PI3K as previously reported. (TIF) [file pone.0039908.s001.tif]
